# Integrating trauma, self-disturbances, cognitive biases, and personality into a model for the risk of psychosis: a longitudinal study in a non-clinical sample

**DOI:** 10.1007/s00406-021-01355-8

**Published:** 2021-12-02

**Authors:** Renata Pionke-Ubych, Dorota Frydecka, Andrzej Cechnicki, Martyna Krężołek, Barnaby Nelson, Łukasz Gawęda

**Affiliations:** 1 Experimental Psychopathology Lab, Institute of Psychology, Polish Academy of Sciences, Jaracza 1, 00-378 Warsaw, Poland; 2 Department of Psychiatry, Wroclaw Medical University, Wroclaw, Poland; 3 Department of Community Psychiatry, Chair of Psychiatry, Medical College Jagiellonian University, Krakow, Poland; 4 II Department of Psychiatry, The Medical University of Warsaw, Warszaw, Poland; 5 Orygen, Parkville, VIC Australia; 6 Centre for Youth Mental Health, The University of Melbourne, Parkville, VIC Australia

**Keywords:** Self-disturbances, Trauma, Cognitive biases, Personality, Psychosis risk

## Abstract

The hypothesis of the psychosis continuum enables to study the mechanisms of psychosis risk not only in clinical samples but in non-clinical as well. The aim of this longitudinal study was to investigate self-disturbances (SD), a risk factor that has attracted substantial interest over the last two decades, in combination with trauma, cognitive biases and personality, and to test whether SD are associated with subclinical positive symptoms (PS) over a 12-month follow-up period. Our study was conducted in a non-clinical sample of 139 Polish young adults (81 females, age *M* = 25.32, SD = 4.51) who were selected for frequent experience of subclinical PS. Participants completed self-report questionnaires for the evaluation of SD (IPASE), trauma (CECA.Q), cognitive biases (DACOBS) and personality (TCI), and were interviewed for subclinical PS (CAARMS). SD and subclinical PS were re-assessed 12 months after baseline measurement. The hypothesized model for psychosis risk was tested using path analysis. The change in SD and subclinical PS over the 12-month period was investigated with non-parametric equivalent of dependent sample t-tests. The models with self-transcendence (ST) and harm avoidance (HA) as personality variables were found to be well-fitted and explained 34% of the variance in subclinical PS at follow-up. Moreover, we found a significant reduction of SD and subclinical PS after 12 months. Our study suggests that combining trauma, cognitive biases, SD and personality traits such as ST and HA into one model can enhance our understanding of appearance as well as maintenance of subclinical PS.

**Supplementary Information:**

The online version contains supplementary material available at 10.1007/s00406-021-01355-8.

## Introduction

The hypothesis of the psychosis continuum, according to which psychotic symptoms have a continuous, non-dichotomous nature and the gradation of their intensity can be observed on a continuum, has been confirmed by numerous empirical studies [1]. The prevalence of psychotic experiences in the general population is estimated at 8% [2]. Most of these experiences are transitory, but in a small percentage of people they may become persistent and lead to clinical disorders [2]. An important and confirmed by various research implication of the hypothesis of the psychosis continuum is the fact that both psychotic disorders and clinical high-risk states as well as psychotic-like experiences (PLEs) occurring in healthy individuals are characterized by similar biological [3–5] and psychological mechanisms [6–10], as well as similar interactions of genetic and psychosocial factors [11]. It enables to study the mechanisms of susceptibility to psychosis among individuals with a high frequency of psychotic experiences who do not suffer from psychotic disorders.

Most people experiencing psychotic symptoms, even with a clinical diagnosis of ultra-high risk (UHR) state, do not develop psychotic disorders in the following years [12]. Therefore, we observe in recent years an intensive search for a combination of early risk factors and mechanisms of psychotic symptom development [13, 14] that would increase the precision of predicting conversion to psychosis.

One of the factors considered by phenomenologically oriented researchers as crucial for understanding the development and the mechanisms of schizophrenia spectrum disorders (SSD) are self-disturbances (SD) [15–17]. These phenomena have a long phenomenological tradition in schizophrenia research as the concept of schizophrenia has been perceived since its very beginning as a disorder of self, which is reflected in its term (*schizo* = split, *phrene* = mind) [18]. SD represent a wide range of anomalous subjective experience that have in common a deformed sense of first-person perspective, which is a deficiency in the sense of being the subject, a self-coinciding center of the action, thought and experience [19].

Studies have shown that SD correlate with positive, negative and disorganized symptoms [20–23], which is in line with theoretical accounts that psychotic symptoms could arise in a response to profound disturbances in the so-called minimal self [15, 24]. They are also frequent in clinical high-risk (CHR) states for psychosis such as attenuated positive symptom syndrome (APS), brief limited intermittent psychotic symptoms (BLIPS) and cognitive basic symptoms (COGDIS) [23, 25, 26]. Moreover, SD were found to be related to the future transition to psychosis in UHR population [27]. More and more often, SD are investigated in disorders beyond SSD, e.g. panic disorder [28], bipolar disorder [29], dissociative disorder [30], borderline personality structure [31]. The presence of SD outside SSD raises questions about their specificity for this group of disorders. Interestingly, the latest meta-analysis [32] indicates their gradient intensity, with the higher severity in SSD as compared to other clinical disorders and the lowest in healthy control groups. Thus, SD can be considered more broadly, not only as phenomena that constitute the core of schizophrenia but also as self-experiences present on a continuum of a varying intensity [32]. This seems to be confirmed by studies revealing their occurrence in non-clinical populations where they correlate with PLEs [33–35] and schizotypy [36, 37]. Some authors also point to the need to search for these types of SD that, apart from their intensity, would distinguish SSD from other disorders [28]. Indeed, the recent study has found that among first-episode psychotic patients SD significantly distinguished between schizophrenia-spectrum and non-schizophrenia spectrum psychoses [38].

SD in SSD are considered a trait-like phenomena that are stable because they reflect an alteration in the structure of consciousness (i.e. structural instability of the ‘minimal’ self) rather than fleeting changes in mental content (which is the case of other symptoms) [39, 40]. The notion of persistence of SD in SSD has gained empirical support [41, 42]. However, their temporal dynamics still need further investigation, also in non-clinical samples, as such studies are very rare. Another aspect of SD that requires further research is associated with factors contributing to the development of these anomalies. Different cognitive and neurocognitive constructs have been proposed to underlie SD, such as source monitoring [43] and aberrant salience [44], abnormalities of the default mode network [45], perceptual incoherence [46], metacognition [47] and cognitive biases [33, 34]. An important role in shaping SD has also been observed in environmental or social stressors such as childhood traumatic events in schizophrenia [48]. Some authors have hypothesized that certain features of SD could appear as a defensive reaction to or an attempt to cope with traumatic circumstances [45]. Indeed, in our previous studies [33, 34] we tested a model of exposure to trauma and cognitive biases contributing to SD in non-clinical samples. Not only exposure to trauma and cognitive biases were significantly associated with SD, but also SD and cognitive biases mediated the relationship between trauma and self-report PLEs.

In the present study, we aimed to expand this model by exploring clinically verified subclinical positive symptoms (PS) as well as personality traits as an additional potential factor that could underlie the formation or maintenance of SD. Personality is a well-recognized time-invariant risk factor for psychopathology, including psychosis [49, 50]. A growing line of research has found that exaggerated maladaptive personality traits (e.g. high harm avoidance and low self-directedness) are related to PLEs [51, 52], the onset of psychosis [53] and relapses [54]. Some studies have also shown that personality traits are related to PLEs through cognitive biases [55, 56]. Altogether, prior studies suggest the possible interplay of personality, cognitive biases and risk for psychosis.

Our sample consists of individuals from the general (non-clinical) population that experience frequent subclinical PS. In the present study, we did not search for SD understood strictly as a nucleus of SSD but rather various phenomena of anomalous subjective experiences that lie on a continuum similar to psychotic experiences. We aimed to extend existing knowledge by combining the described risk factors into one model to enhance our understanding of the risk of psychosis. First, we aimed to test the hypothesized model in which: (1) exposure to trauma, cognitive biases as well as personality traits serve as mechanisms of SD; (2) SD are associated with subclinical PS both at baseline and follow-up assessment. Second, we aimed to investigate the longitudinal course of SD by measuring the construct twice over an interval of 12 months. To the best of our knowledge, our study is the first that combines testing trauma, cognitive biases and personality as the mechanisms of SD with studying explanatory value of SD for subclinical PS in 12-month follow-up.

## Methods

### Participants

The study was organized in three stages. In the first stage, 6264 non-help-seeking Polish young adults (3932 females) from the general population aged between 18 and 35 years (*M* = 26.51, SD = 4.76) were screened via the internet for psychometric risk of psychosis using the Prodromal Questionnaire (PQ-16) [57]. Participants were enrolled from three large Polish cities: Warsaw, Krakow and Wroclaw. Those who scored in the top 7%^1^ on the PQ-16 (i.e. had frequent subclinical psychotic symptoms) and met inclusion criteria were approached to participate in the second stage of the study conducted through face-to-face assessment. Exclusion criteria for participants were screened with self-report questions which included: a history of any psychotic or neurological diagnosis, history of antipsychotic medication treatment and substance dependence disorder in the previous 6 months. Inclusion criteria were met by 438 people, however, 245 respondents could not be contacted or refused to participate in the second stage of the study. The final sample in the second stage of the study consisted of 193 individuals (111 females, age *M* = 25.36, SD = 4.69). Face-to-face baseline assessment in this stage of the study involved assessment of SD, subclinical PS (a structured clinical interview), exposure to traumatic life events, cognitive biases as well as temperament and character. Twelve months after the baseline assessment all 193 participants were contacted and invited to participate in the third stage of the study, that was conducted through face-to-face assessments as well. Follow-up included evaluation of the frequency of SD and subclinical PS since the baseline assessment. Those who were successfully contacted and agreed to take part in the follow-up study formed the final sample consisting of 139 individuals (81 females, age *M* = 25.32, SD = 4.51). The ethics committee of the Medical University of Warsaw approved the study.

### Measures

*Subclinical positive symptoms (PS)* in the screening stage of the study were evaluated with the sixteen-item Prodromal Questionnaire (PQ-16) [57], which operationalizes psychosis risk as a presence of PLEs. Cronbach’s alpha for the total score was 0.82. To investigate subclinical PS in the baseline (PS I) and follow-up (PS II) stage of the study we used the Comprehensive Assessment of At-Risk Mental States (CAARMS) [58]. It is a semi-structured interview designed to identify individuals who are at clinical ultra-high risk (UHR) of developing psychosis. The CAARMS is widely used both in clinical practice and in research on the psychosis risk [59]. It allows to study a wide range of attenuated psychopathology and functioning factors over time. We conducted a full interview, but for the purposes of this study we focused on the results on the positive symptom subscale, to which UHR criteria relate. Cronbach’s alpha for the positive symptom subscale calculated in our sample was 0.81 for baseline and 0.84 for follow-up.

*Self-disturbances (SD)* in the baseline (SD I) and follow-up (SD II) were evaluated with the Inventory of Psychotic-Like Anomalous Self-Experiences (IPASE) [60]. It consists of five dimensions, representing qualitatively different aspects of self-disorder: (1) Cognition (2) Self-Awareness and Presence (3) Consciousness (4) Somatization and (5) Demarcation/Transitivism. Cronbach's alpha for the total score calculated in our sample for both baseline and follow-up was 0.97.

*Exposure to trauma (*psychological, physical, sexual) in the baseline stage was assessed with the Childhood Experience of Care and Abuse Questionnaire (CECA.Q) [61]. Cronbach’s alpha for the total score in our sample was 0.95.

*Cognitive biases* in the baseline stage were measured with the Davos Assessment of Cognitive Biases Scale (DACOBS) [62]. DACOBS consists of seven subscales: jumping to conclusions bias, belief inflexibility bias, attention to threat bias, external attribution bias, social cognition problems, subjective cognitive problems and safety behaviors. Cronbach's alpha for the total score in our sample was 0.88.

*Temperament and character* in the baseline stage were assessed with the Temperament and Character Inventory (TCI) [63]. TCI consists of four temperament subscales: novelty seeking (NS), harm avoidance (HA), reward dependence (RD), and persistence (P) and three character subscales: self-directedness (SDR), cooperativeness (CO), and self-transcendence (ST). Cronbach’s alpha in our sample ranged from 0.51 to 0.82.

A detailed description of all measures can be found in Supplementary Materials.

### Statistical analysis

In the first step, we examined the relationships between potential confounding variables (gender and age) and trauma as the independent variable and subclinical PS at follow-up as the outcome. For this purpose, we conducted correlational analysis for age and Mann–Whitney *U* test for gender.

Next, correlations among all variables were analysed by calculating Spearman’s rank correlation coefficients. The specific associations were then evaluated with structural equation modeling (SEM) in a series of path analyses to test our theoretical model. We tested for the indirect effect of traumatic life events through SD, cognitive biases and TCI to subclinical PS in the follow-up. For this purpose, we used the bootstrap method as recommended by Preacher and Hayes [64]. As we did not have any assumptions with regard to associations between specific personality traits and SD, we ran the model with each particular TCI subscale that turned out to be significantly correlated with SD I and cognitive biases. Due to the complexity of the hypothesized model and moderate sample size we used single-sum indicators instead of latent variables, which enabled increase of stability of the parameter estimates for the models. To control for a history of any psychiatric diagnosis, this variable was included as a covariate in path analyses. Additionally, in Supplementary Materials (Figs. 3–6) we present models separately for baseline and follow-up results.

The goodness of fit to the data for path analyses was estimated with the maximum likelihood estimation procedure with the Bollen-Stine bootstrap (*n* = 2000) procedure of correction for non-normal distribution. We verified goodness of model fit following the guidelines from literature [65]: RMSEA < 0.06 (The Root Mean Square Error of Approximation); SRMR < 0.08 (The Standardized Root Mean Square Residual); CFI > 0.95 (Confirmatory Fit Index) and TLI > 0.95 (Tucker–Lewis Index).

Additionally, we calculated whether the difference in the magnitude of the correlation between our main factors, i.e. SD and subclinical PS at baseline and follow-up was statistically significant. Finally, we conducted the non-parametric Wilcoxon signed-rank test for IPASE and CAARMS positive subscale to compare the results from baseline and follow-up measurement, as well as calculating Cohen’s *d* effect size. Both in the case of correlational analyses and group comparisons we used Benjamini–Hochberg procedure of correction for multiple testing [66].

All statistical analyses were performed with SPSS version 25.0 and Amos version 25.0.

## Results

### Characteristics of the sample

Sample characteristics are presented in Table 1. None of the participants were identified as UHR according to the CAARMS criteria since no one met both the subclinical symptom and functional criteria needed to make such a diagnosis. However, 51 participants at baseline (26.42% of the sample) and 23 participants at follow-up (16.55% of the sample) met the subclinical symptom criterion alone, without a decline in social functioning.

**Table 1:** Sample demographic and characteristics

	*N* (%)		Mean (SD)
Gender		Age	25.32 (4.51)
Male	58 (41.7%)	PQ-16 (screening)	22.98 (4.67)
Female	81 (58.3%)	IPASE I (total score)	140.33 (45.83)
Professional situation		IPASE II (total score)	122.06 (42.70)
Study	70 (50.4%)	CECA.Q (total score)	166.06 (53.76)
Work	98 (70.5%)	Mother antipathy	20.21 (7.20)
Unemployed	4 (2.9%)	Mother neglect	14.89 (5.81)
Rent	2 (1.4%)	Father antipathy	21.33 (8.68)
Education		Father neglect	20.97 (8.22)
Primary	5 (3.6%)	Mother psychological abuse	18.05 (14.60)
Secondary	1 (0.7%)	Father psychological abuse	16.61 (18.01)
Vocational	63 (45.3%)	Role reversal	53.26 (10.48)
Incomplete higher	20 (14.4%)	Physical abuse	0.40 (0.49)
Higher	50 (36.0%)	Sexual abuse	0.34 (0.83)
Psychiatric diagnosis	30 (21.6%)	CAARMS	
Anxiety disorder	18 (12.9%)	Subclinical PS I	9.96 (7.27)
Depression	21 (15.1%)	Subclinical PS II	7.01 (7.38)
Bipolar disorder	1 (0.7%)	DACOBS (total score)	162.60 (26.80)
OCD	1 (0.7%)	Jumping to conclusion	27.22 (5.11)
Eating disorder	3 (2.2%)	Belief inflexibility	18.66 (5.34)
Personality disorder	2 (1.4%)	Attention to threat	27.36 (5.34)
Other	3 (2.2%)	External attribution	22.67 (5.68)
		Social cognition problems	26.29 (6.23)
		Subjective cognitive problems	26.44 (7.11)
		Safety behaviors	13.97 (5.86)
		TCI	
		Harm avoidance	20.81 (8.29)
		Novelty seeking	20.16 (5.92)
		Reward dependence	14.28 (3.56)
		Persistence	4.61 (1.72)
		Self-directedness	19.78 (8.58)
		Cooperativeness	27.65 (8.0)
		Self-transcendence	16.08 (6.82)

*OCD* obsessive–compulsive disorder; *I* measurement at baseline; *II* measurement in 12-month follow-up; *PQ-16* Prodromal Questionnaire-16; *IPASE* Inventory of Psychotic-Like Anomalous Self-Experiences; *CECA.Q* Childhood Experience of Care and Abuse Questionnaire; *CAARMS* Comprehensive Assessment of At-Risk Mental States; *subclinical PS* subclinical positive symptoms; *DACOBS* Davos Assessment of the Cognitive Biases Scale; *TCI* Temperament and Character Inventory

### Correlational analysis

Table 2 shows the detailed results for the relationships between exposure to trauma, cognitive biases, temperament and character as well as SD and subclinical PS both at baseline and follow-up. All the hypothesized relationships turned out to be significant thus allowing for their further investigation in path analysis. With regard to temperament and character four traits were significantly related to SD at baseline and cognitive biases (self-transcendence, harm avoidance, self-directedness and cooperativeness), therefore we ran the model separately for each of these personality traits.

**Table 2:** Correlational analysis

	Trauma	Cognitive biases	SD I	SD II	Subclinical PS I	Subclinical PS II
Trauma						
Cognitive biases	0.20*					
Self-disturbances I	0.29**	0.37***				
Self-disturbances II	0.23**	0.17*	0.59***			
Subclinical PS I	0.05	0.36***	0.34***	0.26**		
Subclinical PS II	0.07	0.20*	0.32***	0.37***	0.46***	
Harm avoidance	0.13	0.42***	0.35***	0.21**	0.24**	0.31***
Novelty seeking	0.12	− 0.09	0.03	− 0.05	− 0.12	− 0.28**
Reward dependence	0.04	− 0.02	− 0.01	− 0.09	0.04	− 0.02
Persistence	− 0.11	− 0.14	− 0.13	− 0.06	− 0.25**	− 0.03
Self-directedness	− 0.27**	− 0.57***	− 0.47***	− 0.27**	− 0.20*	− 0.28**
Cooperativeness	− 0.15	− 0.47***	− 0.29**	− 0.14	− 0.26**	− 0.20*
Self-transcendence	0.02	0.19*	0.30***	0.19*	0.20*	0.15

*SD* self-disturbances; *subclinical PS* subclinical positive symptoms; *I* measurement at baseline; *II* measurement in 12-month follow-up

Coefficients marked in bold were significant after Benjamini–Hochberg correction (*p* < 0.05)

^*^p < 0.05, ** *p* < 0.01, *** *p* < 0.001

The difference between the correlation magnitude of SD I and subclinical PS I at baseline (*r* = 0.34) as well as of SD II and PS II at follow-up (*r* = 0.37) was insignificant (*p* = 0.48).

### Path analyses

The results of Mann–Whitney *U* tests showed no significant difference between men and women in exposure to trauma (*Z* =  − 0.662; *p* > 0.05) and subclinical PS at follow-up (*Z* = −0.623; *p* > 0.05). Age did not correlate with severity of trauma (*r*_s_ = −0.11; *p* > 0.05) or subclinical PS at follow-up (*r*_s_ = −0.13; *p* > 0.05). Therefore, we did not include gender and age in path analyses.

**Fig. 1:**
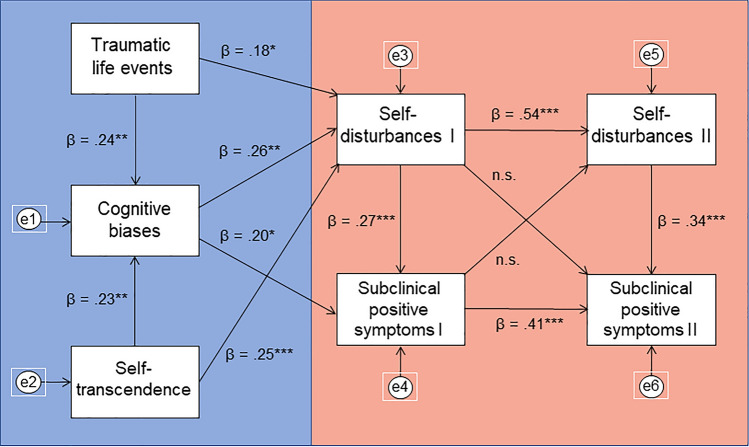
Path analysis with self-transcendence. The model has satisfactory fit indices: (χ^2^ (11) = 5.117, *p* = 0.925; RMSEA = 0.000 [90% CI = 0.000–0.029] *p* = 0.978, CFI = 1.00, TLI = 1.083, SRMR = 0.033). The bootstrapping estimate revealed a significant standardized indirect effect of traumatic life events through all other variables to subclinical positive symptoms II (β = 0.088, 95% CI = 0.043—0.147, *p* = 0.002). This model explained 33.9% of the variance in subclinical positive symptoms II and 33.2% in self-disturbances II. Different colours in the figure mark two parts of the model—one that refers to the mechanisms of self-disturbances and the other that indicates the association of subclinical positive symptoms and self-disturbances in 12-month follow-up based on their baseline measurement. *p < 0.05, **p < 0.01, ***p < 0.001, *n.s.* non-significant

**Fig. 2:**
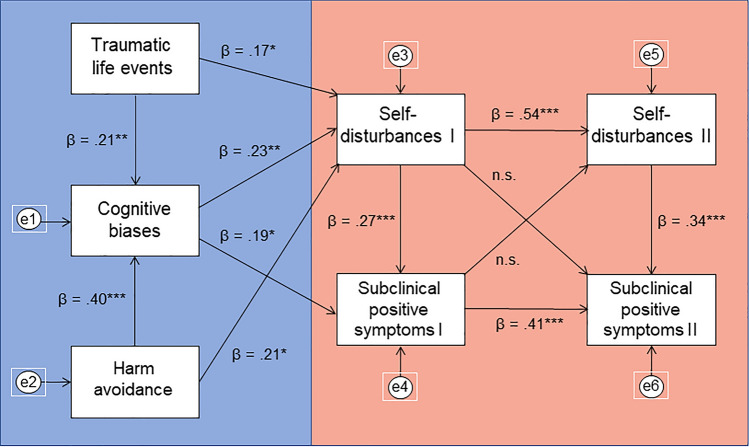
Path analysis with harm-avoidance. Results of path analysis suggested an acceptable model fit: (χ^2^ (11) = 17.701, *p* = 0.089; RMSEA = 0.066 [90% CI = 0.000–0.121] *p* = 0.281, CFI = 0.971, TLI = 0.917, SRMR = 0.062). The bootstrapping estimate revealed a significant standardized indirect effect of traumatic life events through all other variables to subclinical positive symptoms II (β = 0.080, 95% CI = 0.038–0.137, *p* = 0.003). This model explained 33.7% of the variance in subclinical positive symptoms II and 32.9% in self-disturbances II. Different colours in the figure mark two parts of the model—one that refers to the mechanisms of self-disturbances and the other that indicates the association of subclinical positive symptoms and self-disturbances in 12-month follow-up based on their baseline measurement. *p < 0.05, **p < 0.01, ***p < 0.001, *n.s.* non-significant

Results of the first path analysis with self-transcendence (ST) are presented in Fig. 1. The model fit the data well: χ^2^ (11) = 5.117, *p* = 0.925; RMSEA = 0.000 [90% CI = 0.000–0.029] *p* = 0.978, CFI = 1.00, TLI = 1.083, SRMR = 0.033. However, the paths from SD I to subclinical PS II and from subclinical PS I to SD II were not significant. The second path analysis with harm avoidance (HA) is shown in Fig. 2. This model had the following fit indices: χ^2^(11) = 17.701, *p* = 0.089; RMSEA = 0.066 [90% CI = 0.000–0.121] *p* = 0.281, CFI = 0.971, TLI = 0.917, SRMR = 0.062. The RMSEA and TLI fit indices were slightly below a very good level of fit, however, still acceptable. As with the first model, the path from SD I to subclinical PS II and from subclinical PS I to SD II were insignificant. Next, we run the model with self-directedness, which had acceptable fit, however, multiple paths turned out to be insignificant (from trauma to SD I and cognitive biases, from cognitive biases to SD I as well as from SD I to subclinical PS II and from subclinical PS I to SD II). The last model included cooperativeness. Likewise, it had a satisfactory model fit, but insignificant paths from cooperativeness to SD, as well as the cross paths between SD and subclinical PS at baseline and follow-up. The last two models are presented in Supplementary Materials (Figs. 1, 2).

### Wilcoxon signed-rank test

Result of the Wilcoxon signed-rank test for each dimension of SD as well as for subclinical PS are presented in Table 3. There were significant moderate correlations between both SD and subclinical PS at baseline and follow-up, with moderate to large significant reductions of all symptoms over the 12-month follow-up period.

**Table 3:** Result of the Mann–Whitney *U* tests and correlational analysis

	Baseline *M* (SD)	Follow-up *M* (SD)	*Z*	Cohen’s *d*	Spearman’s *r*_*s*_
IPASE total score	140.33 (45.83)	122.06 (42.70)	− 5.105***	0.961	0.59***
Cognition	16.09 (6.36)	13.54 (5.69)	− 4.799***	0.891	0.50***
Self-awareness and presence	52.98 (19.15)	45.99 (17.32)	− 4.715***	0.873	0.60***
Consciousness	18.75 (5.74)	16.60 (5.65)	− 4.227***	0.768	0.49***
Somatization	41.56 (14.74)	36.05 (13.69)	− 4.540***	0.835	0.56***
Demarcation/Transitivism	10.96 (4.27)	9.88 (3.98)	− 3.441**	0.610	0.55***
CAARMS subclinical PS	9.96 (7.27)	7.01 (7.38)	− 4.620***	0.852	0.46***

*IPASE* Inventory of Psychotic-like Aomalous Self-Experiences; *CAARMS* Comprehensive Assessment of at-Risk Mental States; *subclinical PS* subclinical positive symptoms

^**^p < 0.01, ***p < 0.001

## Discussion

Our study was conducted within the framework of the psychosis continuum hypothesis. Hence, we have investigated the subclinical psychotic symptoms and their potential psychological mechanisms among those participants who have had relatively frequent subclinical psychotic symptoms and have never been diagnosed or suffering from full-blown psychotic symptoms. To the best of our knowledge, this is the first study to jointly investigate interrelationships between exposures to traumatic life events, cognitive biases and personality traits as potential mechanisms driving SD as well as its explanatory value for the level of subclinical PS at baseline and 12-month follow-up. The main finding of our study is that combining personality traits, namely harm avoidance and self-transcendence, together with a history of exposure to traumatic-life events, cognitive biases and SD into one integrated model of psychosis risk significantly explained the frequency and severity of subclinical PS 12 months from the initial assessment.

We found a significant indirect effect of trauma on subclinical PS, which is in line with a consistent body of research on the importance of early traumatic events in shaping psychosis risk [67–71]. Our results revealed this relationship as indirect, supporting the notion that experience of trauma alone may not be enough to increase the likelihood of the onset of psychosis and other factors likely mediate this path [72]. We hypothesized that these other factors that could be directly impacted by trauma and which could then contribute to the formation of subclinical PS are cognitive biases and SD. Thus, in our model we assumed that to generate subclinical PS trauma first needs to disrupt the minimal self and to distort cognitive information processing of the environment. As pointed out by Sass and Borda [73], the reason for the relationship between trauma and SD may lie in dissociative reactions. They postulated that these dissociative processes may be a defensive reaction to social stressors such as traumatic environmental circumstances, describing this as a form of *secondary* diminished self-presence, which is one of the key aspects of SD [45]. Indeed, associations between trauma and SD as well as between trauma and dissociative processes have been found in clinical [48, 74] and non-clinical samples [33, 34, 75]. On the other hand, the relationship between trauma and cognitive biases that we found in the current study is consistent with other research showing that traumatic life events negatively affect information processing [33, 75–77]. Our results are also in line with theoretical considerations on the triangle of the interrelations between trauma, cognitive biases and psychotic symptoms [78, 79]. Based on cognitive models of psychosis [80, 81] it is hypothesized that exposure to early social adversities such as childhood neglect or abuse may bias cognitive schemas in a way that encourages individuals to perceive the world as threatening and hostile, thus concentrating on searching for sources of potential harm and attributing negative events to external factors outside of their control. These distorted cognitive schemas contribute to the interpretation of experiences with psychotic explanations [78]. Moreover, it was postulated by Nelson et al. [43] that some neurocognitive processes could be considered as correlates of SD. A recent study has shown that source monitoring deficits, which is a cognitive bias involving difficulties in making attribution about origins of experience, is associated with SD in patients with early psychosis [82]. Results of our study indicate that this relationship applies also to other dysfunctional patterns of information processing that are measured by the DACOBS.

In our model, we assumed that personality traits create the basis for the formation of cognitive biases and not the other way around, based on studies showing heritability not only of temperamental traits but also of character [83]. The decision on this direction of the relationship between cognitive biases and personality was dictated also by the results of previous studies [55, 56]. In path analyses HA and ST turned out to be associated with both cognitive biases and SD. These traits have consistently been reported to be elevated in patients with schizophrenia [84–90] (also in remission [91]), their first-degree relatives with schizotypal features [92], UHR individuals [49] and in non-clinical samples where it correlated with schizotypal traits [93, 94]. With regard to HA, there is evidence that this personality feature may be considered a schizophrenia-related endophenotype marker [86, 88, 95]. The underlying reason for the association between HA and cognitive biases seems to be especially clear when it comes to safety behaviors and their most common type—avoidance of threat. Engaging in behaviours that aim at avoiding potential harm and reducing perceived interpersonal threat seems to be motivated by anxiety that is a core substance of HA. This is consistent with previous studies showing associations between safety behaviours and anxiety [96]. In addition, anxiety is related to lower cognitive performance and difficulties with concentration [97] which could explain the association between HA and another cognitive bias—subjective cognitive problems. Concerning the path from HA to SD, we hypothesize that a link could possibly exist between pessimistic worry, which is a component of HA [98], and hyperreflexivity as one of the aspects of SD. People with a high level of HA are characterized by excessive, unnecessary worrying and dwelling on problems which potentially can contribute to exaggerated self-consciousness and heightened awareness of aspects of one’s experience.^2^ As for ST, its association with cognitive biases can be understood through external attribution and subjective cognitive problems. People with high levels of ST tend to be absent-minded, thus can have problems with task focus and can perceive themselves as having cognitive difficulties. On the other hand, beliefs in forces that are outside of one’s control may lead to making external attributions of unpleasant events. Furthermore, the connection between ST and SD could stem from the notion that high ST involves the dissolution of clear boundaries between self and others and the sense of unity with the world [99]. It could be assumed that a high level of this trait predisposes to dissociative processes such as depersonalization, which is a one possible manifestation of SD. Dissociation is also related to transitivistic phenomena that constitute one of the domains of SD. As a matter of fact, ST has previously been found to be associated with self-reported dissociation [100, 101]. Furthermore, in a study conducted by Park et al. [102] in a UHR sample, ST was associated with what the authors called a pre-reflective self factor that consisted of basic symptoms, magical ideation and perceptual aberration. In another study by Boeker et al. [90], ST was found to be related in patients with schizophrenia to ego pathology, which is a construct resembling SD.

It is worth noting that none of the study participants met both symptom and functional criteria necessary for a diagnosis of UHR. Our group functioned well socially and professionally, which does not seem to be surprising, as they were recruited from the general population that was not seeking help. However, 51 participants (26.42% of the sample) at baseline and 23 participants (16.55% of the sample) at follow-up experienced subclinical PS at a level that allowed symptom criterion to be met. These subclinical PS turned out to be mostly transient, since we found a significant decrease at a 12-month follow-up. According to existing knowledge it is the expected result. Various studies have observed a similar decrease in psychotic symptoms over time [103–106], which seems to be a part of their natural course and recovery processes [107]. Meta-analysis conducted by Fusar-Poli et al. [12] has shown that most of the UHR patients do not progress towards psychosis, i.e. subclinical symptoms often decline with time. However, in some individuals in the general population they may persist and develop into clinical disorders [2]. Our correlational analysis between subclinical PS and SD at baseline and follow-up showed that, even though these phenomena decrease over time, the level of their relatedness remain very similar.

Moreover, we found a significant reduction also in SD and all of its domains, which seems to contradict theoretical [39] and empirical [41, 42, 108] accounts on their trait-like nature. However, those studies were conducted in clinical or help-seeking samples and our research is the first to investigate the longitudinal course of SD in a non-clinical sample from the general population. As SD in healthy individuals, even with high-frequent subclinical PS, are less pronounced than in SSD, they may be more susceptible to fluctuation and its appearance may depend more on external factors such as social stressors. Similarly to Svendsen et al. [109, 110], who found a decrease in SD in a seven-year follow-up study, and in accordance with theoretical accounts [73], it is possible that the decline in SD in our sample could be due mostly to a change in *secondary* SD, i.e. defensive-compensatory response to external stressors. *Secondary* SD may be more prone to change and may cease with the disappearance of stressful factors [45]. We can hypothesize that the cessation of stressors between baseline and follow-up may lea d to a reduction both in SD and subclinical PS. Interestingly, Kelleher et al. [111] in their prospective study of childhood trauma showed that the cessation of trauma resulted in a reduced incidence of psychotic experiences. Additionally, it is possible that a high level of resources, such as good functioning, protected people in our sample from the perpetuation of subclinical PS and SD, and from seeking help because of them. Social impairment is considered to be an important predictor of a long-term outcome [59].

The decrease in SD and PS (potentially in response to the cessation of stressors) could also partly explain why two of the paths in our model were nonsignificant, mainly from SD I to subclinical PS II and from subclinical PS I to SD II. Our results show that subclinical PS and SD are clearly interrelated, but only at the same time of measurement. As SD in our study did not show high temporal persistence and we assume that in our sample they were mostly *secondary* and state-like, then their appearance could be related to subclinical PS but only at the same time point. It is, therefore, possible that early exposure of trauma, cognitive biases as well as ST and HA jointly predispose to the formation of SD, however, their appearance (at least *secondary* SD) is triggered mostly by external factors such as present environmental stressors. Moreover, the fact that we have found SD to be present outside SSD, in a non-clinical sample, seems to indicate that they lie on a continuum similar to PLEs. Indeed, a recent meta-analysis conducted by Raballo et al. [32] showed that have a gradient of severity, i.e. higher severity in SSD than in clinical groups outside SSD and healthy control groups.

There are several limitations to the present research. Although our study involved re-measurement of SD and subclinical PS at 12-month follow-up, it cannot establish causality. As the study design did not involve experimental manipulation we cannot preclude that between the first and second measurement other factors associated with the change in subclinical PS came into play. Moreover, since our participants functioned well socially and professionally and were not seeking help, taking into consideration protective factors such as resilience, coping strategies and stress sensitivity would be of importance in future studies. It should also be noted that although subclinical PS were evaluated via structured interview, which allowed to reduce false-positive results, this was not the case for SD, for which we used a self-report questionnaire. This type of measurement is limited when it comes to assessing a complex construct such as SD [112] and does not allow for clinical validation of these experiences. Future research should use comprehensive structured clinical interviews such as the EASE [19] and the EAWE [113] as well as objective experimental measures that do not rely on participant insight (e.g. the sense of agency task [114]). Another limitation of our study is a lack of clinical evaluation of personality disorders, especially borderline features, that are associated with experiencing PLEs [115], and could have confounded our results. Future studies could address this issue by including e.g. the Assessment of DSM-IV Personality Disorders questionnaire (ADP-IV). Additionally, it is worth noting that there was still 66% of the variance in subclinical PS not explained by variables in our models, thus future research should take into account other risk factors, e.g. polygenic risk score. Furthermore, our model was tested in a sample of non-clinical psychosis risk, consisting of individuals who were not seeking help and were not recruited from clinical settings, which is worth noting in the light of risk enrichment research [116]. Thus, the results should not be directly translated into populations at clinical risk of psychosis and future studies should address this issue by testing the model in UHR sample. Another issue to be addressed is the relatively high but expected (21.6%) prevalence of psychiatric diagnoses (anxiety and depressive in particular) in our sample, which probably affected our results. However, comorbidity in psychotic disorders and its risk states is a common phenomenon [12, 105, 106]. With no doubt there is an interrelationship between anxiety and psychosis [117] and these phenomenon are hard to separate in research practice. We think, however, that controlling psychiatric diagnoses in the analyses make our results more clear.

It is worth mentioning that those individuals from online screening with whom we could not contact may potentially have a higher psychosis risk. We conducted analyses comparing those who met the inclusion criteria and were invited to face-to-face study, but did not agree to take part in it or it was impossible to contact them (e.g. they did not answer the phone, hung up, etc.) with the people who participated. There was no significant difference in terms of PLEs on the screening tool PQ-16. The issue of losing participants appeared also at the final stage of the study. Twenty-eight percent of the baseline sample was not successfully contacted or refused to participate in the follow-up. Those participants did not significantly differ from the final sample in terms of baseline assessments of subclinical PS, SD, trauma, cognitive biases, HA, ST or social functioning. However, we cannot exclude the possibility that they differed in other characteristics, that we had not taken into account, but could have an impact on the final results.

Last but not least, the threshold we adopted to recruit individuals from online screening to face-to-face study obviously impacted obtained results. We chose to select people within the highest percentage of PLEs. All of these individuals met the Ising criterion [57], i.e. confirmed the presence of at least 6 PLEs in the PQ-16. However, other strategies are also possible, e.g. looking for a natural cut-off in the distribution of PLEs.

To conclude, our study suggests that combining trauma, cognitive biases, SD and personality traits such as ST and HA into one model can enhance our understanding of the appearance as well as maintenance of subclinical PS. Future studies should verify how targeting cognitive biases and SD in early interventions, for example with the use of Metacognitive Reflection and Insight Therapy (MERIT) [118] and metacognitive training (MCT) [119], impact the frequency and intensity of subclinical PS.

## Supplementary Information

Below is the link to the electronic supplementary material.

Supplementary file1 (DOCX 320 KB)

## Data Availability

Data can be requested from the corresponding author.

## Footnotes

1 We aimed to recruit approximately 200 participants from approximately 6000 subjects (3.3% of the sample studied) who would obtain scores on the PQ-16 within the top 10%. We chose a wider percentage of the highest scores to recruit from, expecting that not all participants would meet the inclusion criteria and would be willing to participate in the second stage of the study. Finally, we assessed 193 people whose results on the PQ-16 against the entire sample turned out to be in the top 7%.

2 It should be noted that reverse direction is also possible, i.e. hyperreflexivity contributes to increasing HA.
