# Supplementary material for: Integrating trauma, self-disturbances, cognitive biases, and personality into a model for the risk of psychosis: a longitudinal study in a non-clinical sample
Source: Eur Arch Psychiatry Clin Neurosci. 2021 Dec 2;272(6):1073–85. doi: 10.1007/s00406-021-01355-8 (PMC9388435; doi:10.1007/s00406-021-01355-8)
Supplement: Supplementary file 1 — Supplementary file1 (DOCX 320 KB) [file 406_2021_1355_MOESM1_ESM.docx]

*European Archives of Psychiatry and Clinical Neuroscience*

**Integrating trauma, self-disturbances, cognitive biases, and personality into a model for the risk of psychosis. A longitudinal study in a non-clinical sample.**

Renata Pionke-Ubych, Dorota Frydecka, Andrzej Cechnicki, Martyna Krężołek, Barnaby Nelson, Łukasz Gawęda^*^

*Corresponding author:

Łukasz Gawęda, Ph.D., Institute of Psychology, Polish Academy of Sciences, Jaracza 1, 00-378 Warsaw, Poland, email: l.gaweda@psych.pan.pl

**SUPPLEMENTARY MATERIAL**

**Measures**

*Subclinical positive symptoms (PS)*

For evaluation of subclinical psychotic symptoms in the screening stage of the study we used the sixteen-item Prodromal Questionnaire (PQ-16) (Ising *et al.*, 2012). The PQ-16 is a self-report questionnaire to screen for psychosis risk operationalized as a presence of psychotic-like symptoms. It is a shortened version of the 92-item PQ (Loewy *et al.*, 2005) and consists of items that assess perceptual abnormalities and hallucinations, unusual thought content, delusional ideas, and paranoia as well as negative symptoms on a scale: present vs. non-present (true vs. false) which we modified to better reflect the frequency of subclinical psychotic symptoms. Specifically, we used a four-point scale: ‘never’, ‘sometimes’, ‘often’ and ‘almost always’. Most of the items in the PQ-16 refer to attenuated positive psychotic symptoms. The PQ-16 has satisfactory psychometric characteristics in the assessment of subclinical psychotic symptoms with a specificity and sensitivity of 87% in discriminating patients meeting the criteria of UHR from those who do not meet UHR criteria (Ising *et al.*, 2012). We used a Polish version of the questionnaire (Gaweda *et al.*, 2018a). Cronbach’s alpha for the total score was 0.82.

To investigate subclinical PS in the baseline (PS I) and follow-up (PS II) stage of the study we used the Polish version (Jaracz *et al.*, 2012) of the Comprehensive Assessment of At-Risk Mental States (CAARMS) (Yung *et al.*, 2005). The CAARMS is a semi-structured interview designed to investigate different aspects of attenuated psychopathology and functioning factors over time. The CAARMS consists of 7 subscales: positive symptoms (subclinical delusions and hallucinations); negative symptoms; cognitive changes; behavior changes; motor or physical changes; emotional disturbances; general psychopathology. Symptoms are evaluated for their severity and frequency on scales ranging from 0 to 6. In our study, we focused on the severity and frequency of the positive symptom subscale that includes: unusual thought content, non-bizarre ideas, perceptual abnormalities, and disorganized speech. Cronbach’s alpha for the positive symptom subscale calculated in our sample was 0.81 for baseline and 0.84 for follow-up.

*Self-disturbances (SD)*

For evaluation of SD in the baseline (SD I) and follow-up (SD II) stage of the study we used the Inventory of Psychotic-Like Anomalous Self-Experiences (IPASE) (Cicero *et al.*, 2017), which is a 57-item self-report questionnaire based on the phenomenological description of self-disorders in schizophrenia spectrum disorders. The IPASE is similar to the gold-standard assessment of the self-disturbances - The Examination of Anomalous Self Experience (EASE) (Parnas *et al.*, 2005) interview as the IPASE was developed to include similar concepts to the EASE. However, the IPASE items were developed independently and for that reason the IPASE should be considered an independent measure rather than a self-report version of existing clinical interviews (Cicero *et al.*, 2016). The items are clustered into five dimensions, representing qualitatively different aspects of self-disorder: 1) Cognition (e.g. ‘I feel like my thoughts are being generated by someone else.’); 2) Self-Awareness and Presence (e.g. ‘I feel as though I no longer have an identity.’) ; 3) Consciousness (e.g. ‘I have difficulty telling whether I am experiencing something or just imagining it’); 4) Somatization (e.g. ‘I have had the feeling that I am watching myself from outside of my body.’) and 5) Demarcation/Transitivism (e.g. ‘I wonder whether or not I truly exist.’). The scale has been used in studies on psychosis proneness (Cicero *et al.*, 2017) and among schizophrenia spectrum patients (Cicero *et al.*, 2016). We used a Polish version of the IPASE (Gaweda *et al.*, 2018b). Cronbach's alpha for the total score calculated in our sample for both baseline and follow-up was 0.97.

*Exposure to trauma*

To assess exposure to trauma in the baseline stage of the study we used the Childhood Experience of Care and Abuse Questionnaire (CECA.Q) (Smith *et al.*, 2002). It is a self-report measure that investigate traumatic life events retrospectively such as lack of parental care (neglect and antipathy), parental psychological abuse, role reversal, parental physical abuse, and sexual abuse from an adult before the age of 17. The CECA.Q has been validated among psychotic patients (Fisher *et al.*, 2011). It consists of different types of trauma subscales that covers a wide assessment of traumatic life events. Cronbach’s alpha for the total score in our sample was 0.95.

*Cognitive biases*

To measure cognitive biases in the baseline stage of the study we used the Davos Assessment of Cognitive Biases Scale (DACOBS) (van der Gaag *et al.*, 2013). The 42-item DACOBS consists of seven subscales and three clusters related to different types of biases: (1) specifically associated with psychosis: jumping to conclusions bias, belief inflexibility bias, attention to threat bias, external attribution bias, (2) associated with cognition: social cognition problems and subjective cognitive problems, and (3) related to coping strategies: safety behaviors. The total score indicates the overall severity of cognitive biases. The Polish version of the DACOBS was prepared with a back-translation procedure and has been used previously (Gaweda *et al.*, 2018b, Gawęda *et al.*, 2015b). Cronbach's alpha for the total score in our sample was 0.88.

*Temperament and character*

To assess temperament and character in the baseline stage of the study we used the Temperament and Character Inventory (TCI) (Cloninger *et al.*, 1994). The TCI is a self-report measure with 240 statements which can be answered as ‘true’ or ‘false’. The questionnaire address Cloninger’s psychobiological model of temperament and character (Cloninger *et al.*, 1993). The temperament dimension consists of four subscales: novelty seeking (NS) – which refers to the tendency towards excitation in response to novel stimuli; harm avoidance (HA) – the tendency to inhibit behavior in response to negative stimuli; reward dependence (RD) – the tendency to maintain or continue behavior in response to social reward; and persistence (P) – the tendency to persevere in a behavior despite frustration and fatigue. The character dimension consists of three subscales: self-directedness (SD) – which refers to the extent to which an individual is responsible, goal-oriented and self-confident; cooperativeness (CO) – which refers to the extent to which individuals conceive themselves as integral parts of human society; and self-transcendence (ST) – which is the ability to conceive oneself as an integral part of the universe as a whole. We used the Polish version of the TCI which has acceptable psychometric properties (Cronbach’s alphas ranging from 0.50 for the P subscale to 0.90 for the HA subscale) (Hornowska, 2003). The scale was used by authors in previous studies on psychotic-like experiences in a healthy population. Cronbach’s alpha in our sample ranged from 0.51 to 0.82.

**Figure 1. Path analysis with self-directedness**

**
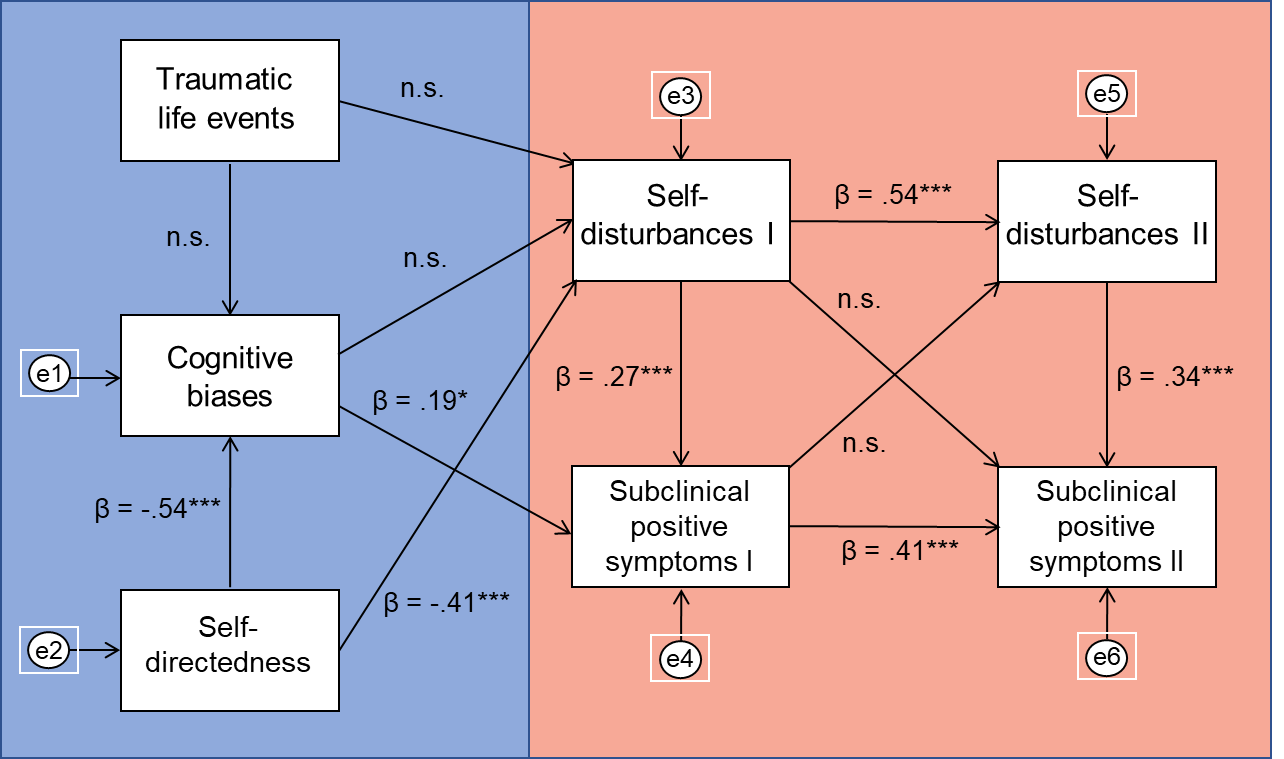
**

*Note*: Results of path analysis suggested a model with acceptable fit: (χ2 (11) = 17.245, p = 0.101; RMSEA = 0.064 [90% CI = 0.000–0.119] p = 0.304, CFI = 0.974, TLI = 0.934, SRMR = 0.0601). Different colours in the figure mark two parts of the model – one that refers to the mechanisms of self-disturbances and the other that indicates the prediction of subclinical positive symptoms and self-disturbances in 12-month follow-up based on their baseline measurement. This model explained 33.6% of the variance in subclinical positive symptoms II and 32.5% in self-disturbances II.

* p < 0.05, *** p < 0.001, n.s., non-significant

**Figure 2. Path analysis with cooperativeness**


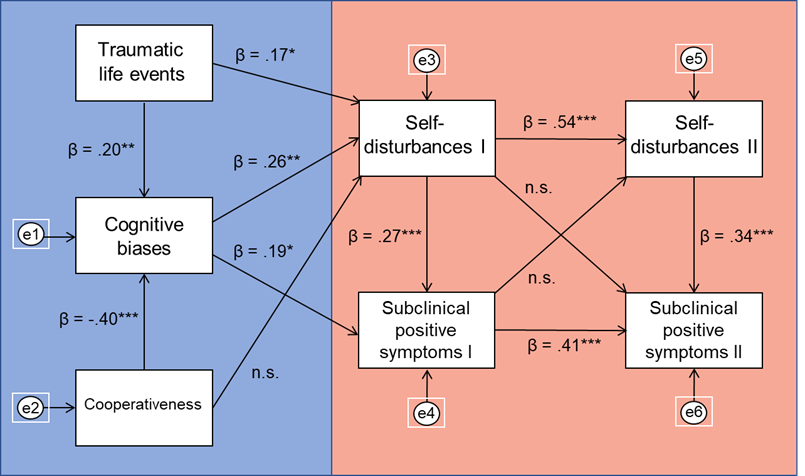


*Note:* Results of path analysis suggested a model that fit the data well: (χ2 (11) = 7.036, p = 0.796; RMSEA = 0.000 [90% CI = 0.000–0.059] p = 0.926, CFI = 1.000, TLI = 1.052, SRMR = 0.0399). The bootstrapping estimate revealed a significant standardized indirect effect of traumatic life events through all other variables to subclinical positive symptoms II (β = 0.080, 95% CI = 0.035 - 0.138, p = 0.002). This model explained 33.8% of the variance in subclinical positive symptoms II and 33.2% in self-disturbances II. Different colours in the figure mark two parts of the model – one that refers to the mechanisms of self-disturbances and the other that indicates the prediction of subclinical positive symptoms and self-disturbances in 12-month follow-up based on their baseline measurement.

* p < 0.05, ** p < 0.01, *** p < 0.001, n.s., non-significant

**Figure 3. Path analysis with self-transcendence and baseline measures of self-disturbances and subclinical positive symptoms**


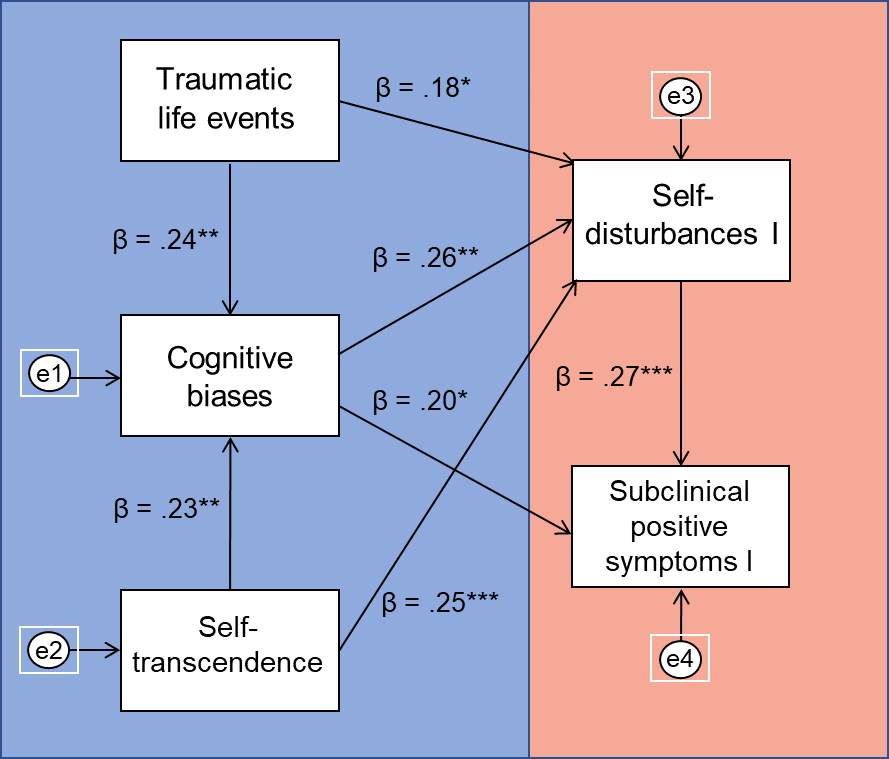


*Note:* Results of path analysis suggested a model that fit the data well: (χ2 (5) = 3.655, p = 0.600; RMSEA = 0.000 [90% CI = 0.000–0.101] p = 0.754, CFI = 1.000, TLI = 1.052, SRMR = 0.039). This model explained 20.3% of the variance in subclinical positive symptoms II and 23.3% in self-disturbances II.

* p < 0.05, ** p < 0.01, *** p < 0.001, n.s., non-significant

**Figure 4. Path analysis with self-transcendence and follow-up measures of self-disturbances and subclinical positive symptoms**


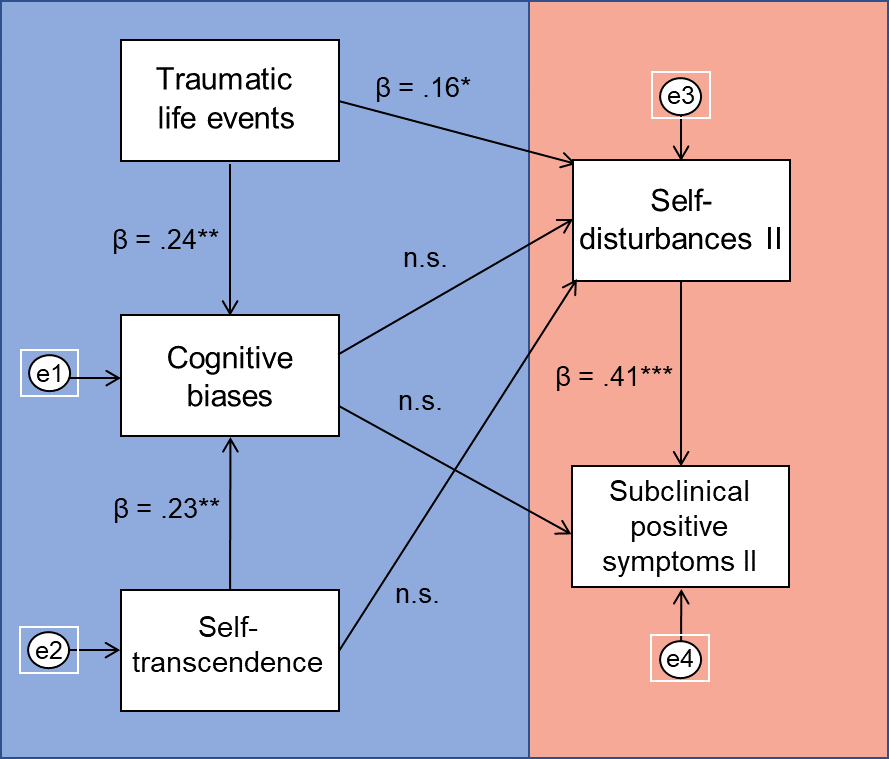


Note: Results of path analysis suggested a model that fit the data well: (χ2 (5) = 2.763, p = 0.736; RMSEA = 0.000 [90% CI = 0.000–0.085] p = 0.850, CFI = 1.000, TLI = 1.131, SRMR = 0.033). This model explained 20.0% of the variance in subclinical positive symptoms II and 9.0% in self-disturbances II.

* p < 0.05, ** p < 0.01, *** p < 0.001, n.s., non-significant

**Figure 5. Path analysis with harm avoidance and baseline measures of self-disturbances and subclinical positive symptoms**


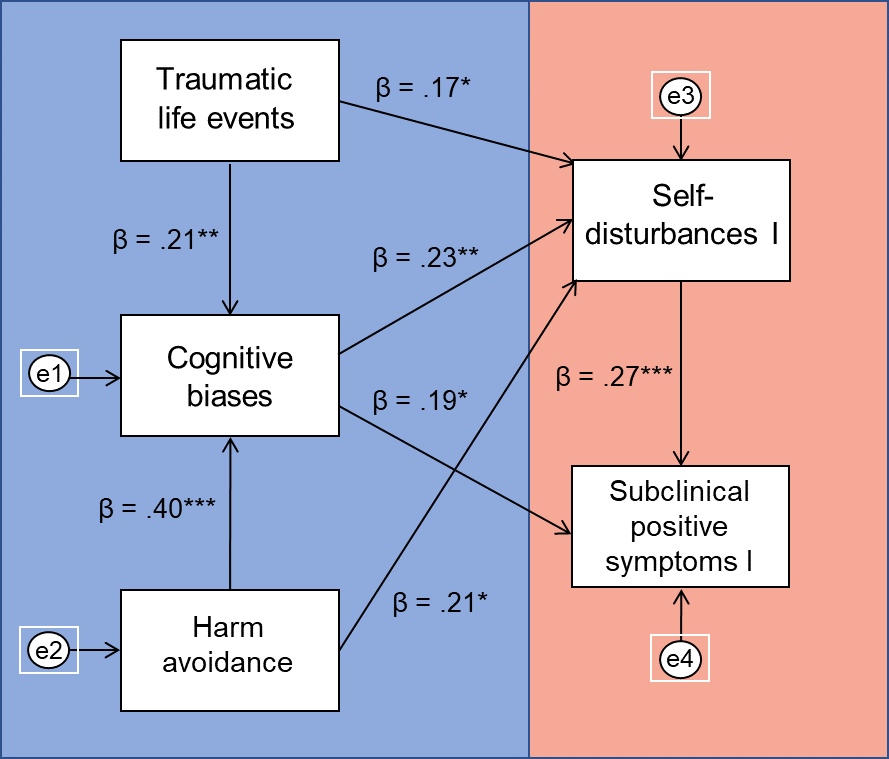


Note: Results of path analysis suggested a model with unsatisfactory fit: (χ2 (5) = 10.428, p = 0.064; RMSEA = 0.089 [90% CI = 0.000–0.165] p = 0.167, CFI = 0.944, TLI = 0.832, SRMR = 0.065). This model explained 20.0% of the variance in subclinical positive symptoms II and 19.8% in self-disturbances II.

* p < 0.05, ** p < 0.01, *** p < 0.001, n.s., non-significant

**Figure 6. Path analysis with harm avoidance and follow-up measures of self-disturbances and subclinical positive symptoms**


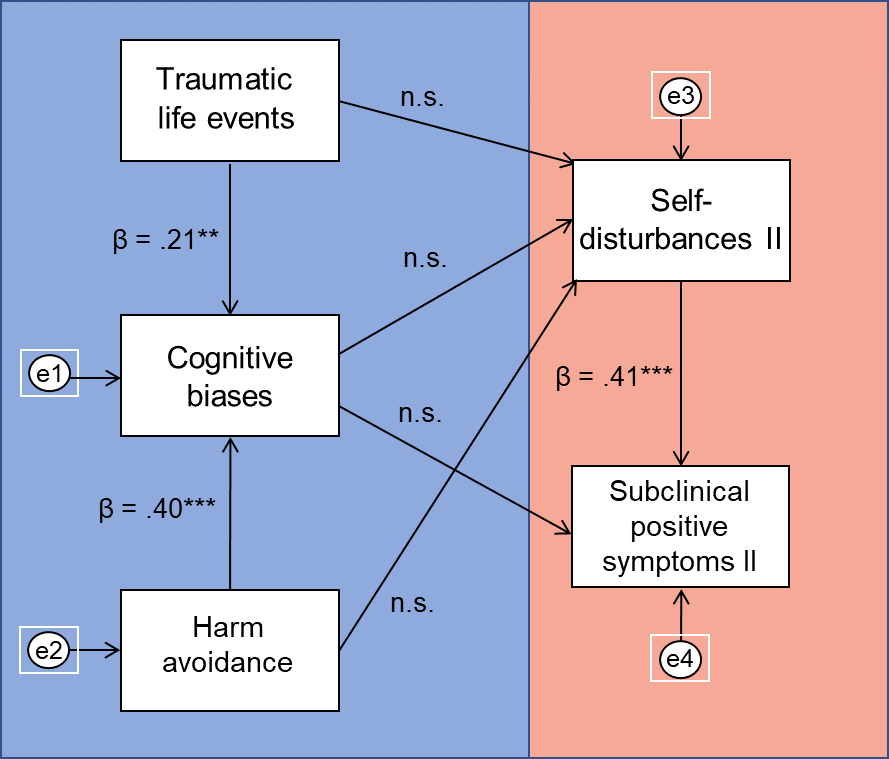


Note: Results of path analysis suggested a model with unsatisfactory fit: (χ2 (5) = 15.585, p = 0.008; RMSEA = 0.124 [90% CI = 0.057–0.196] p = 0.037, CFI = 0.868, TLI = 0.605, SRMR = 0.073). This model explained 19.8% of the variance in subclinical positive symptoms II and 8.3% in self-disturbances II.

* p < 0.05, ** p < 0.01, *** p < 0.001, n.s., non-significant

**References:**

**Cicero, D. C., Klaunig, M. J., Trask, C. L. & Neis, A. M.** (2016). Anomalous self-experiences and positive symptoms are independently associated with emotion processing deficits in schizophrenia. *Schizophrenia Research* **176**, 456-461.

**Cicero, D. C., Neis, A. M., Klaunig, M. J. & Trask, C. L.** (2017). The Inventory of Psychotic-Like Anomalous Self-Experiences (IPASE): Development and validation. *Psychological Assessment* **29**, 13.

**Cloninger, C. R., Przybeck, T. R., Svrakic, D. M. & Wetzel, R. D.** (1994). The Temperament and Character Inventory (TCI): A guide to its development and use. *Washington University: Centre for Psychobiology of Personality, St. Louis, Missouri*.

**Cloninger, C. R., Svrakic, D. M. & Przybeck, T. R.** (1993). A psychobiological model of temperament and character. *J Archives of General Psychiatry* **50**, 975-990.

**Fisher, H. L., Craig, T. K., Fearon, P., Morgan, K., Dazzan, P., Lappin, J., Hutchinson, G., Doody, G. A., Jones, P. B. & McGuffin, P.** (2011). Reliability and comparability of psychosis patients’ retrospective reports of childhood abuse. *Schizophrenia Bulletin* **37**, 546-553.

**Gaweda, L., Pionke, R., Krezolek, M., Prochwicz, K., Klosowska, J., Frydecka, D., Misiak, B., Kotowicz, K., Samochowiec, A., Mak, M., Bladzinski, P., Cechnicki, A. & Nelson, B.** (2018a). Self-disturbances, cognitive biases and insecure attachment as mechanisms of the relationship between traumatic life events and psychotic-like experiences in non-clinical adults - A path analysis. *Psychiatry Res* **259**, 571-578.

**Gaweda, L., Prochwicz, K., Adamczyk, P., Frydecka, D., Misiak, B., Kotowicz, K., Szczepanowski, R., Florkowski, M. & Nelson, B.** (2018b). The role of self-disturbances and cognitive biases in the relationship between traumatic life events and psychosis proneness in a non-clinical sample. *Schizophr Res* **193**, 218-224.

**Gawęda, Ł., Prochwicz, K. & Cella, M.** (2015b). Cognitive biases mediate the relationship between temperament and character and psychotic-like experiences in healthy adults. *Psychiatry Research* **225**, 50-57.

**Hornowska, E.** (2003). Temperamentalne uwarunkowania zachowania [Temperamental determination of behavior]. *Poznań: Bogucki Wydawnictwo Naukowe*.

**Ising, H. K., Veling, W., Loewy, R. L., Rietveld, M. W., Rietdijk, J., Dragt, S., Klaassen, R. M., Nieman, D. H., Wunderink, L., Linszen, D. H. & van der Gaag, M.** (2012). The validity of the 16-item version of the Prodromal Questionnaire (PQ-16) to screen for ultra high risk of developing psychosis in the general help-seeking population. *Schizophr Bull* **38**, 1288-96.

**Jaracz, J., Grzechowiak, M., Raczkowiak, L., Rataj, K. & Rybakowski, J.** (2012). Polish version of Comprehensive Assessment of At Risk Mental States (CAARMS)--the description of the method. *Polish Psychiatry* **46**, 95-107.

**Loewy, R. L., Bearden, C. E., Johnson, J. K., Raine, A. & Cannon, T. D.** (2005). The prodromal questionnaire (PQ): preliminary validation of a self-report screening measure for prodromal and psychotic syndromes. *Schizophrenia Research* **79**, 117-125.

**Parnas, J., Moller, P., Kircher, T., Thalbitzer, J., Jansson, L., Handest, P. & Zahavi, D.** (2005). EASE: Examination of Anomalous Self-Experience. *Psychopathology* **38**, 236-58.

**Smith, N., Lam, D., Bifulco, A. & Checkley, S.** (2002). Childhood Experience of Care and Abuse Questionnaire (CECA.Q). Validation of a screening instrument for childhood adversity in clinical populations. *Soc Psychiatry Psychiatr Epidemiol* **37**, 572-9.

**van der Gaag, M., Schutz, C., Ten Napel, A., Landa, Y., Delespaul, P., Bak, M., Tschacher, W. & de Hert, M.** (2013). Development of the Davos assessment of cognitive biases scale (DACOBS). *Schizophr Res* **144**, 63-71.

**Yung, A. R., Yuen, H. P., McGorry, P. D., Phillips, L. J., Kelly, D., Dell'Olio, M., Francey, S. M., Cosgrave, E. M., Killackey, E., Stanford, C., Godfrey, K. & Buckby, J.** (2005). Mapping the onset of psychosis: the Comprehensive Assessment of At-Risk Mental States. *Aust N Z J Psychiatry* **39**, 964-71.
